# Influence of ERβ selective agonism during the neonatal period on the sexual differentiation of the rat hypothalamic-pituitary-gonadal (HPG) axis

**DOI:** 10.1186/2042-6410-3-2

**Published:** 2012-01-19

**Authors:** Heather B Patisaul, Sandra M Losa-Ward, Karina L Todd, Katherine A McCaffrey, Jillian A Mickens

**Affiliations:** 1 Department of Biology, North Carolina State University, Raleigh, NC 27695, USA

**Keywords:** hypothalamus, development, sex differences, estrogen, kisspeptin

## Abstract

**Background:**

It is well established that sexual differentiation of the rodent hypothalamic-pituitary-gonadal (HPG) axis is principally orchestrated by estrogen during the perinatal period. Here we sought to better characterize the mechanistic role the beta form of the estrogen receptor (ERβ) plays in this process.

**Methods:**

To achieve this, we exposed neonatal female rats to three doses (0.5, 1 and 2 mg/kg) of the ERβ selective agonist diarylpropionitrile (DPN) using estradiol benzoate (EB) as a positive control. Measures included day of vaginal opening, estrous cycle quality, GnRH and Fos co-localization following ovariectomy and hormone priming, circulating luteinizing hormone (LH) levels and quantification of hypothalamic kisspeptin immunoreactivity. A second set of females was then neonatally exposed to DPN, the ERα agonist propyl-pyrazole-triol (PPT), DPN+PPT, or EB to compare the impact of ERα and ERβ selective agonism on kisspeptin gene expression in pre- and post-pubescent females.

**Results:**

All three DPN doses significantly advanced the day of vaginal opening and induced premature anestrus. GnRH and Fos co-labeling, a marker of GnRH activation, following ovariectomy and hormone priming was reduced by approximately half at all doses; the magnitude of which was not as large as with EB or what we have previously observed with the ERα agonist PPT. LH levels were also correspondingly lower, compared to control females. No impact of DPN was observed on the density of kisspeptin immunoreactive (-ir) fibers or cell bodies in the arcuate (ARC) nucleus, and kisspeptin-ir was only significantly reduced by the middle (1 mg/kg) DPN dose in the preoptic region. The second experiment revealed that EB, PPT and the combination of DPN+PPT significantly abrogated preoptic Kiss1 expression at both ages but ARC expression was only reduced by EB.

**Conclusion:**

Our results indicate that selective agonism of ERβ is not sufficient to completely achieve male-typical HPG organization observed with EB or an ERα agonist.

## Background

The relative mechanistic roles the two primary forms of the estrogen receptor (ERα and ERβ) play in the estrogen-dependent organization of the hypothalamic-pituitary-gonadal (HPG) axis remain incompletely characterized. The present study addressed this data gap by assessing the impact of ER-selective agonism during neonatal life on HPG organization. Within the HPG axis, reproductive maturation and function is largely coordinated by the release of gonadotropin releasing hormone (GnRH) [1,2]. In the adult, GnRH secretion is regulated by positive and negative steroid feedback loops. These hormone sensitive, neuroendocrine pathways are sexually dimorphic and organized, primarily, by hormones in a series of perinatal critical periods. In adult females, a "surge" of GnRH, elicited through positive feedback by the pre-ovulatory rise of estradiol, induces the release of luteinizing hormone (LH) and, consequently, ovulation. In male rodents, neonatal estrogen, aromatized from testicular androgen, acts within the HPG axis to defeminize the male hypothalamus such that GnRH neurons do not respond to elevated estrogen levels with a surge of GnRH. Similarly, neonatal estrogen administration can also defeminize the female hypothalamus resulting in the inability to generate a GnRH surge in adulthood [3].

We have previously shown that neonatal administration of estradiol benzoate (EB) or the ERα selective agonist propyl-pyrazole-triol (PPT) to female rats results in the premature onset of anestrus and, following ovariectomy (OVX) and hormone priming, little to no Fos labeling in GnRH neurons (an indicator of GnRH activation) [4,5]. These effects signify the incapacitation of the steroid positive feedback system. In contrast, while neonatal administration of the ERβ selective agonist diarylpropionitrile (DPN) also impacted estrous cyclicity and GnRH activation to some degree, the magnitude of each effect was considerably smaller compared to EB or PPT administration [4]. For example, most DPN exposed females ultimately developed an irregular estrous cycle, but this occurred several weeks after females exposed to PPT or EB became anestrus, most of which were acyclic within days of pubertal onset. GnRH and Fos co-labeling was approximately 41% lower in the DPN exposed animals compared to control females, but this decline was not statistically significant. Collectively, these results could indicate that either (1) the dose of DPN used in our prior study was insufficient to produce a maximal effect, or (2) that ERβ plays a less critical mechanistic role than ERα in the defeminization of GnRH signaling pathways. The first experiment in the present study was undertaken to definitively resolve this issue by more comprehensively examining the relative role ERβ might play in the sex specific organization and function of the HPG axis.

Although GnRH neurons express ERβ, and thus can potentially respond to DPN directly, it is generally accepted that hormonal signals are largely coordinated by other estrogen responsive neurons within the hypothalamus, which then convey this information to the GnRH neurons [3,6]. One group of neurons that has recently emerged as critical regulators of GnRH secretion are those which produce kisspeptin (Kiss), a family of proteins coded for by the Kiss1 gene. There is rapidly emerging evidence that Kiss neurons directly innervate GnRH neurons and are vital for the regulation of GnRH secretion in many species, including humans [7-12]. The kisspeptin receptor (Kiss1r, formerly GPR54) is constitutively expressed in GnRH neurons [13-16]. Activation of this receptor was demonstrated to be required for the initiation of puberty with the discovery that humans or mice with a mutated form of Kiss1r fail to enter puberty and remain hypogonadal [17,18].

In the rat, there are two major populations of Kiss neurons, a preoptic population spanning the anteroventral periventricular nucleus (AVPV) through the medial preoptic area (MPOA), and one in the arcuate nucleus (ARC). The density of Kiss neurons appears to be sexually dimorphic only in the AVPV, with females having significantly more Kiss neurons, Kiss1 expression and Kiss immunoreactive (-ir) neuronal fibers than males [19-22]. This population is thought to be essential for steroid positive feedback and the initiation of the pre-ovulatory GnRH surge [7,9,13,23-25]. The importance of neonatal estrogen for male-typical organization of AVPV Kiss neurons has been demonstrated by us and others. Neonatal administration of aromatizable androgen [26] reduces Kiss1 expression, while EB or the ERα selective agonist PPT produces male-like levels of Kiss-ir in the rat female AVPV [4,20]. This defeminizing influence of estrogen during the neonatal period is consistent with what is currently known about the structural organization of the AVPV and the sex specific organization of other neuronal populations contained within it [3,27,28]. By contrast, a role for ERβ in the sexual differentiation process has not been clearly established. In the first experiment, using multiple doses of the ERβ agonist DPN, we sought to more definitively determine whether or not neonatal activation of ERβ could also defeminize Kiss-ir in the hormone primed, ovariectomized female rat. A subsequent experiment was then performed to compare how DPN, PPT or the co-administration of both influences the peripubertal ontogeny of Kiss1 expression in gonadally intact females to obtain a comprehensive picture of how ER selective agonism impacts the kisspeptin system across the lifespan.

The ARC population of Kiss neurons is hypothesized to be important for the regulation of steroid negative feedback and energy balance [22,26,29]. Potential sex differences within this population remain incompletely characterized. The density of ARC Kiss-ir fibers is clearly greater in post-pubertal females than males [30]. We have shown that neonatal administration of EB significantly reduces Kiss-ir in the female rat ARC suggesting that estrogen plays an important role in organizing this sex difference. Interestingly, although it was initially shown that the number of Kiss-1 expressing neurons and the density of Kiss1 expression in the ARC is not sexually dimorphic in adults [26] recent work has suggested that the influence of steroid hormone-independent factors on the function of these neurons is sexually dimorphic during peripuberty [31]. This sex difference is most likely attributable to known sex differences in the tempo of pubertal maturation in rodents, with males maturing later than females. This observation indicates that the ARC population may be functionally dimorphic to some degree and thus influenced by neonatal estrogens. We recently revealed robust sex differences in ARC Kiss1 expression prior to weaning (but not after), supporting the hypothesis that neonatal estrogens have the potential to alter the sex specific organization of the ARC Kiss system [4,20]. We have previously demonstrated that neonatal administration of the ERα selective agonist PPT results in only a marginal decrease in ARC Kiss-ir fibers, suggesting that agonism of ERα alone is insufficient to recapitulate the effect of EB [4,20]. Similarly, neonatal administration of DPN at 1 mg/kg did not appreciably affect the density of ARC Kiss-ir fibers [4]. The plexus of the Kiss-ir fibers in the rat ARC is dense, making neuron number difficult to ascertain. Thus, the impact of estrogens on the number of Kiss neurons has not been clearly established. Moreover, the relative roles ERα and ERβ play in the organization of the ARC Kiss1 population remain undefined. The present experiments fill this data gap by characterizing the effects of DPN, PPT, or the co-administration of both, on Kiss1 mRNA and Kiss-ir in the ARC.

DPN is an ERβ selective agonist with a 70-fold greater relative binding affinity and 170-fold greater relative potency in transcription assays for ERβ than ERα [32]. The doses selected for the present study, 0.5, 1 and 2 mg/kg body weight (designated LOW, MID and HIGH respectively), bracket the 1 mg/kg dose employed in our prior study [4]. They are also well within the range of what other investigators have used to evaluate the functional role of ERβ in other estrogen sensitive neuroendocrine pathways *in vivo *[33-36] and thus below a dose that might generate activity through ERα. We hypothesized that DPN would have a dose dependent effect on HPG de-feminization with the most significant effects produced by the highest dose.

## Methods

### Experiment 1

#### Animal husbandry, neonatal exposure and surgery

Timed pregnant Long Evans rats (n = 10; Charles River, NC, USA) were individually housed in polysulfone caging within a humidity- and temperature-controlled room with a 14-h light, 10-h dark cycle (lights on from 7:00 to 21:00) at 23°C and 50% average relative humidity at the Biological Resource Facility at North Carolina State University (NCSU). Because standard lab chows are soy-based and thus contain significant amounts of phytoestrogens [37-39], all of the animals were fed a phytoestrogen-free diet *ad libitum *for the duration of the experiment (Test Diet 5K96, Purina). The dams littered over three days and each day all newly arrived litters were cross-fostered to minimize litter effects and culled to a maximum litter size of 12. All of the pups within a cross-fostered litter were then assigned to the same treatment group to prevent cross contamination. Each treatment group contained two cross-fostered litters and all animals were treated, regardless of sex. Males remained in the litter but were not used for this study.

Beginning on the day of birth, neonates were subcutaneously (sc) injected with vehicle (0.05 ml, Sigma, St. Louis, MO, USA), estradiol benzoate (EB, 10 μg, Sigma), or one of three doses of the ERβ selective agonist diarylpropionitrile (DPN; 0.5, 1 or 2 mg/kg bw, Tocris Biosciences, Ellisville, MS, USA). These doses were designated LOW, MID, and HIGH respectively. All compounds were dissolved in ethanol, then sesame oil at a ratio of 10% EtOH and 90% oil as we have done previously [27]. The vehicle was also prepared with this ratio. We have found this vehicle to cause less skin irritation than the alternative vehicle, DMSO. The 1 mg/kg dose of DPN is approximately equivalent to what has previously been used by us and others *in vivo *[33,36,40-44]. The other two doses were selected to bracket this dose. The range was narrow but the highest dose was not anticipated to be high enough to activate ERα [33-36]. DPN is an ERβ selective agonist with a 70-fold greater relative binding affinity and 170-fold greater relative potency in transcription assays for ERβ than ERα [32]. The animals (n = 6 to 12 females per group) received injections daily from the day of birth (postnatal day (PND) 0) through PND 3 (four injections total).

All pups were weaned into same sex litter-mate pairs on PND 21, ear punched and maintained on the same light cycle as the dams. Upon weaning, the animals were checked every morning for day of vaginal opening (DOV), a hallmark of puberty in the rat. Because DOV can be accelerated by increased body weight, all animals were weighed on a single day after most animals had undergone vaginal opening (all animals PND 29 or 30 on the day of weighing) to ensure body weights were equivalent. Monitoring of the estrous cycle by vaginal lavage [45] commenced approximately two weeks after vaginal opening. Samples were collected on three consecutive mornings every other week beginning on the predicted day of estrus. We opted to sample every other week to reduce handling stress and minimize the potential for inducing a pseudopregnancy. All animals observed to have stopped cycling (defined by the failure to sequentially progress through proestrus, estrus and diestrus) were reexamined (for three to five consecutive days) two weeks later to confirm that the failure to cycle was persistent and not due to a pseudopregnancy. Sampling proceeded until PND 115.

Animals were ovariectomized (OVX) under isoflurane anesthesia (completed over four days beginning on PND 150) and recovered for three weeks. The OVX females were then injected sc with 10 μg EB dissolved in sesame oil at 0900 h, followed 48 hours later by a sc injection of 500 μg progesterone dissolved in the same vehicle to stimulate a GnRH surge. Animals were then sacrificed by transcardial perfusion (4% paraformaldehyde) six to eight hours after the progesterone injection at the onset of the dark cycle (as we have done previously [4,5]) because this is the point at which GnRH activity peaks using this experimental paradigm [46,47]. Blood was collected by cardiac puncture prior to the onset of perfusion. Brains were removed, post-fixed in 20% sucrose paraformaldehyde, cryoprotected overnight in potassium phosphate buffer containing 20% sucrose, rapidly frozen in powdered dry ice, and stored at -80°C. Brains were then sliced into 35 μm coronal sections, and divided into two series of free-floating alternating sections. Tissue was stored in antifreeze solution (20% glycerol, 30% ethylene glycol in KPBS) at -20°C until immunohistochemical processing.

Animal care, maintenance, surgery and sacrifice were conducted in accordance with the applicable portions of the Animal Welfare Act and the U.S. Department of Health and Human Services "Guide for the Care and use of Laboratory Animals." All experimental procedures involving animals were approved by the NCSU Institutional Animal Care and Use Committee.

#### Immunohistochemistry

A subset of animals was used for the immunostaining (n = 5 per group). From each of these animals, one set of coronal sections comprising the organum vasculosum of the lamina terminalis (OVLT) through the caudal border of the anterior ventral periventricular nucleus (AVPV) were immunolabeled for GnRH and Fos or Kiss using immunohistochemistry (IHC) methods described in detail elsewhere [4,20,48]. Briefly, the first set of tissue was incubated in a cocktail of primary antibodies directed against GnRH (raised in rabbit, 1:8,000, and generously gifted by Dr. Robert Benoit of McGill University Health Center, Montreal, QC, Canada) and Fos (raised in goat, 1:250, Santa Cruz Biotechnology, Santa Cruz, CA, USA; SC-52-6) followed by the secondary antibodies Alexa-Fluor donkey anti-rabbit 488 and Alexa-Fluor donkey anti-goat 555 at 1:400. The second set of tissue was incubated in a primary antibody directed against kisspeptin-10 [49] (raised in rabbit, 1:4,000, and generously gifted by Alain Caraty of Institut National de la Recherche Agronomique/Centre National de la Recherche Scientifique, Université de Tours, France) and the Alexa-Fluor donkey anti-rabbit 488 secondary antibody at 1:400. After secondary antibody incubation, sections were rinsed, mounted onto slides (Superfrost Plus, Fisher, Pittsburgh, PA, USA), and cover-slipped with glycerol mountant (50% glycerol in 4 M sodium bicarbonate).

#### Quantification of GnRH and Fos immunoreactivity

GnRH activation was assessed by quantifying the percent of GnRH neurons co-localized with the early gene product Fos (cFos), a method which is a reliable indicator of GnRH activation [50] following OVX and hormone priming. GnRH/Fos double-immunofluorescent label was visualized and quantified as described in our prior publications [4,5]. GnRH immunoreactive (-ir) neurons were distributed laterally and dorsally to the third ventricle throughout the OVLT and anterior AVPV, as has been described previously [51]. Three sequential sections per animal comprising the OVLT through the midlevel region of the AVPV (corresponding to 0.24 through -0.12 mm from Bregma) were selected for analysis using a brain atlas for reference [52]. Only tissue sets showing consistent GnRH and Fos immunostaining throughout all sections were used for the analysis. They were photographed with a Retiga 1800 monochrome camera (QImaging, Austin, TX USA) attached to a Leica 5000DM microscope (Leica Microsystems, Wetzlar, Germany) fitted with 20× and 40× objective lenses and filter cubes for Cy3 and FITC. The images of each label (GnRH and Fos) were then merged using the MCID Elite Image Analysis (Interfocus Imaging Ltd., Cambridge, England, UK) software package. Single labeled GnRH cells, and cells immunolabeled for both GnRH and Fos were then hand-counted by an individual blind to the treatment groups and verified by a second independent observer. As expected [51,53], the average number of GnRH cells counted per animal did not statistically differ between groups.

#### Quantification of Kiss immunoreactivity in the AVPV and ARC

In mice, Kiss immunoreactivity (-ir) has previously been shown to be localized to extended lengths of fibers throughout the AVPV and within cell bodies located in the medial and caudal AVPV [21]. These cell bodies are readily visible in mice but not in rats (point of discussion, 1^st ^World Conference on Kisspeptin Signaling in the Brain) unless an intracerebroventricular infusion of colchicine is administered [54]. To overcome this technical hurdle, we have previously found that, in the rat, quantitative assessment of AVPV Kiss-ir neuronal fibers is a suitable and reliable alternative to counting Kiss-ir cell bodies [4,20,30] and developed a method for doing so by co-opting techniques used previously to quantify neuronal fiber density in other brain regions [55,56]. This approach does not account for all Kiss immunolabeling present in the entire region of interest but instead gives a representative assessment of the density of labeling within the region. Thus, for the present study, in the AVPV we quantified Kiss-ir fiber density.

In the ARC, Kiss perikarya are sparse but readily labeled in the rat, along with a dense plexus of Kiss-ir fibers [21,30,57]. Cell numbers do not appear to be sexually dimorphic, but the density of Kiss-ir fibers is [19,30]. Thus, for the present study, in the ARC we quantified both Kiss-ir fiber density and cell bodies using confocal microscopy. The total number of Kiss-ir voxels (for fiber density) and Kiss-ir perikarya was calculated for each animal.

To assess the density of AVPV Kiss-ir fibers projecting rostrally from the AVPV to the OVLT GnRH neurons, two to three rostral AVPV sections per animal were selected using a brain atlas [52,58] (corresponding to 0.0 through 0.48 mm from Bregma). In the ARC, six sequential sections comprising the rostral, middle and caudal regions of the ARC (corresponding to -1.92 through -3.36 mm from Bregma) were selected from each animal. Kiss-ir was visualized on a Leica TCS SPE confocal microscope fitted with a 63× oil corrective objective lens. For each scan, a set of serial image planes (z-step distance = 1 μm) was collected through the entire thickness of the section. Individual images were acquired sequentially for light emitted from each fluorophore and parsed into separate stacks of images for analysis using the Image J software package (National Institutes of Health (NIH), Bethesda, MD, USA) as previously described [48]. To control for variations in tissue thickness that would result in unequal numbers of image planes, substacks of consecutive image planes that excluded the rostral and caudal edges of the tissue sections were created for each set of scans. Substacks consisted of 23 sequential image planes for the AVPV and 24 sequential images planes for the ARC. As we have described previously [20,48], individual images contained within each substack were depixelated and binarized to minimize the inclusion of background fluorescence. Fibers were then skeletonized to a thickness of one pixel to compensate for differences in individual fiber thickness and brightness. The number of resulting bright pixels in each image plane was then quantified using the Image J Voxel Counter plug-in (NIH) and summed over the substack. The ARC substacks were then reviewed one at a time and Kiss-ir cell bodies were counted by hand. The resulting number of Kiss-ir voxels and Kiss-ir cell bodies is, therefore, a quantitative representation of Kiss-ir density within the volume sampled but is not meant to be an absolute quantification of the number of Kiss-ir fibers (or cell bodies) contained within the region of interest.

#### Serum LH analysis

Upon collection, blood samples were immediately spun down in a refrigerated centrifuge for 10 minutes at 13,000 rpm. The plasma fraction (n = 5 per group) was then removed and sent to the Biomarkers Core Lab at the Yerkes National Primate Research Center at Emory University (Atlanta, GA, USA) for analysis by radioimmunoassay (RIA). The limit of detection was 1 ng/ml and the intra-assay coefficient of variance was less than 10%. Animals with LH levels below the limit of detection were assigned a level of 1 ng/ml for the statistical analysis.

### Experiment 2

#### Neonatal exposure and tissue collection

To validate the Kiss data obtained from IHC, and to determine approximately how early in development significant effects are discernable, a second set of animals was exposed and used to quantify Kiss1 mRNA levels. Timed pregnant LE rats were obtained (n = 10) to generate pups for Experiment 2, cross-fostered, culled and maintained under the same conditions as described in Experiment 1. For this experiment, only the mid-level dose of DPN was used, a group receiving 1 mg/kg PPT and a group receiving both DPN and PPT (1 mg/kg each) were included to determine if the co-administration of these compounds could replicate the masculinizing effect of EB. Thus, females were exposed to vehicle, EB, MID DPN, PPT (1 mg/kg) or DPN+PPT (1 mg/kg of each) by sc injection from PNDs 0 to 3. The animals were then sacrificed by rapid decapitation on PND 24 (prior to pubertal onset) or 33 (just after pubertal onset) and the brains rapidly removed and flash frozen on dry ice then stored at -80°C. For each brain, serial sections (20 μm thick) comprising the anterior border of the OVLT through the caudal aspect of the ARC (as defined in Experiment 1) were cut and slide mounted using a cryostat. We have previously shown that sex differences in Kiss1 mRNA are appreciable at this age in the AVPV but not the ARC [30].

#### In situ hybridization for Kiss1 mRNA

Kiss1 mRNA levels were assessed using an *in situ *hybridization (ISH) protocol described by us previously [22,30]. Briefly, the cDNA transcriptional template generated to probe Kiss1 mRNA was a 318-bp cDNA insert (Genbank Accession number NM_181692.1) prepared by using reverse transcription-polymerase chain reaction (RT-PCR) with a forward primer of 5'-TCTCCTCTGTGTGGCCTCTT-3' and a reverse primer of 5'-AGGCCAAAGGAGTTCCAGTT-3'. Following ISH, slides were exposed to Kodak Biomax MR X-ray film (Eastman Kodak, Rochester, NY, USA) for 11 days (AVPV) or 24 days (ARC) then developed using an automatic processor (Konica Corporation, Tokyo, Japan). 14C-labeled autoradiographic standards (Amersham Pharmacia Biotech, Piscataway, NJ, USA)) were included in the cassettes for quantification. X-ray film images viewed using a monochrome QICAM 1394 12-bit camera (QImaging, Surry, BC, Canada) mounted above a light-box (supplied by InterFocus Imaging Ltd., Cambridge, England, UK). Relative levels of Kiss1 mRNA in the AVPV and ARC were assessed by optical density from the film autoradiograms using the digital densitometry application of the MCID Core Image software program (InterFocus Imaging Ltd., Cambridge, England, UK) following procedures similar to what we and others have described previously [22,59-61]. For each region of interest (AVPV or ARC), a sampling template encompassing the region of interest was created and used for all sections to standardize the area examined. Brain regions and background levels were measured unilaterally from anatomically matched sections. For each animal, three measurements per region of interest were obtained (one each from the rostral, medial and caudal regions) along with an unlabeled background area at each point. The three values obtained for each animal were then averaged to obtain a representative measurement for that animal. Average background levels were then subtracted to obtain a final value. Optical densities were converted to nCi/g tissue equivalents using autoradiographic 14C microscales (Amersham Life Sciences, Arlington Heights, IL, USA).

#### Statistical analysis

For Experiment 1, DOV, body weight, plasma LH levels, the percent of GnRH and FOS co-labeled cells, the number of ARC Kiss-ir cells, and the density of voxels containing Kiss immunolabeling within the OIL, EB, LOW DPN, MID DPN and HIGH DPN groups were compared by one-way analysis of variance (ANOVA) with exposure group as the factor and followed up with Fisher's Least Significant Difference (LSD) *post-hoc *tests for individual comparisons when appropriate (SYSTAT, Systat Software, Inc., Chicago, IL, USA). For Experiment 2, Kiss1 mRNA levels were first compared by two-way ANOVA with age and exposure group as factors, and then followed up with the appropriate one-way ANOVA and Fisher's LSD *post-hoc *test. In all cases, comparisons were two-tailed and the significance level was set at *P *≤ 0.05.

## Results

### Experiment 1

#### Age and weight at vaginal opening

**Figure 1:**
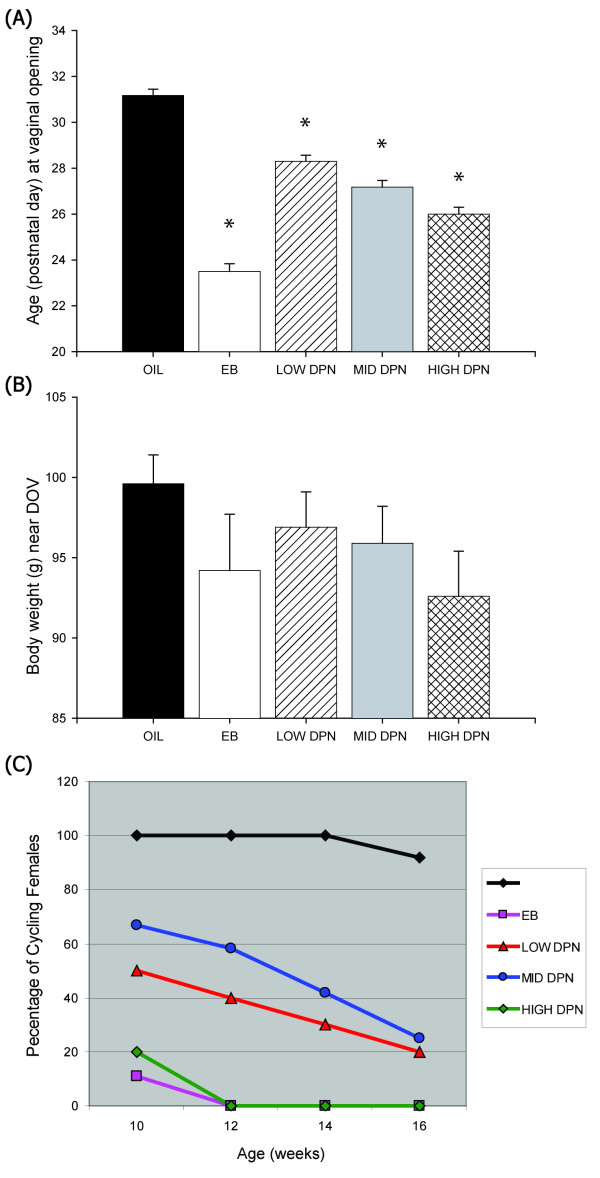
**Effects on reproductive development**. **(A) **Day of vaginal opening (DOV), a hallmark of pubertal onset in the female rat, was significantly advanced by EB and all doses of DPN compared to the vehicle treated controls. **(B) **There was no overall effect of treatment on bodyweight near the time of vaginal opening. **(C) **By 10 weeks of age, nearly all of the females neonatally administered EB or the 2 mg/kg dose of DPN (HIGH DPN) were acyclic while all of the vehicle control animals (Oil) were cycling normally. By 16 weeks of age, 75% of the MID DPN treated females and 85% of the LOW DPN treated females were acyclic compared to only one of the control females. (Means ± S.E.M., *P ≤ 0.001).

There was a significant effect of exposure on DOV (F(4,45) = 82.63, *P *≤ 0.001). Compared to the vehicle treated controls (n = 12), DOV occurred approximately a week earlier in the females neonatally exposed to EB (Figure 1A, n = 6, *P *≤ 0.001). DOV was also significantly advanced by neonatal exposure to DPN in a dose dependent fashion, with the DOV occurring earliest in the group that received the highest dose (HIGH DPN, n = 10, *P *≤ 0.001). MID DPN advanced DOV by about four days (n = 12, *P *≤ 0.001) and LOW DPN advanced DOV by approximately three days (n = 10, *P *≤ 0.001). There was no significant effect of treatment on body weight (Figure 1B) around the time of DOV (F(4,45) = 1.259, P = 0.30), a result which indicates that weight was not a confounding factor.

#### Estrous cycle data

Regular estrous cycles (Figure 1C) commenced in all exposure groups except the group neonatally exposed to EB. As expected [4,62], EB treated females (eight of nine) failed to enter a normal estrous cycle and instead displayed persistent estrus or diestrus. The one EB female who initially began cycling entered a state of anestrus within two weeks. Neonatal exposure to DPN also resulted in the premature onset of irregular or persistent estrus but the age of onset differed by dose. Loss of a regular estrous cycle occurred earliest in the HIGH DPN group (n = 10, Figure 1C), with only 20% of these females progressing through all stages at 10 weeks of age. In contrast, all of the vehicle treated control females (n = 12) were displaying regular estrus cycles at this age but only 68% (8 of 12) of the MID DPN females and half of the LOW DPN females (5 of 10) were still cycling normally. By 16 weeks of age, none of the EB or HIGH DPN treated females were cycling compared to all but one of the control females (92%), 25% of the MID DPN females and 20% of the LOW DPN females.

#### Co-localization of GnRH and Fos in the OVLT

**Figure 2:**
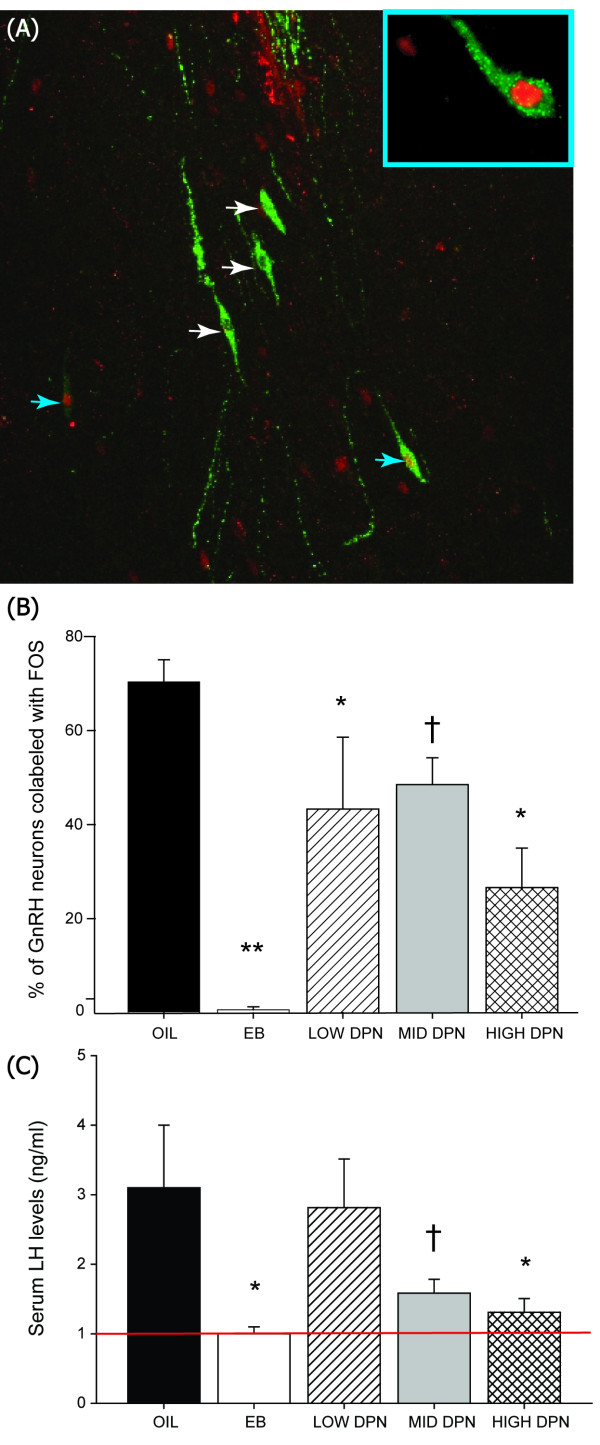
**GnRH activation and LH release following hormone priming**. **(A) **Immunolabeling of GnRH (green) and FOS (red) in the region surrounding the OVLT following hormone priming (Experiment 1). Representative double labeled cells are indicated by the blue arrows and single labeled GnRH neurons are indicated by the white arrows. Double labeled cells were first visualized at low magnification and then verified at higher magnification (inset). **(B) **The percentage of double labeled neurons was significantly reduced by neonatal DPN administration but the magnitude of the effect was substantially greater in the EB treated group. **(C) **LH levels were below the limit of detection (indicated by the red line, 1 ng/ml) in all females neonatally exposed to EB, and three of the five animals in the HIGH DPN group. LH levels were significantly lower in the EB and HIGH DPN groups compared to the vehicle treated control group (OIL, *P *≤ 0.05). A trend for lower LH levels in the MID DPN group was also observed (*P *≤ 0.06). The overall pattern of serum LH levels was largely reflective of GnRH and FOS co-labeling. (3v = 3^rd ^ventricle, Means ± S.E.M., *P ≤ 0.05, **P ≤ 0.001, † < 0.07).

GnRH activation in response to the sequential administration of EB and progesterone was assessed by quantifying the percent of GnRH and Fos co-labeling. There was a significant effect of exposure group (F(4,17) = 10.029, *P *≤ 0.001). Hormone administration successfully induced Fos-ir in 70% of GnRH neurons in the vehicle treated control females (n = 5, Figure 2). In contrast, less than 1% of GnRH neurons in EB treated females were co-labeled with Fos (n = 4, *P *< 0.001) demonstrating the potent defeminizing effect of EB on this endpoint. Compared to the control females, GnRH activation was lower in all of the DPN treated groups (Figure 2B), the magnitude of which was most pronounced at the highest dose (n = 5, *P *≤ 0.001). None of the DPN doses successfully replicated the magnitude of the EB effect. The marginal effect of the 1 mg/kg dose (n = 4, *P *= 0.07) is consistent with, and nearly identical in magnitude to, what we have reported in a past study [4].

#### Serum LH levels

Circulating LH levels (Figure 2C) were significantly influenced by exposure group (F(4,20) = 2.91, *P *≤ 0.04). All of the animals (n = 5 per group) neonatally exposed to EB, and three of the five animals in the HIGH DPN group had LH levels below the limit of detection (1 ng/ml). LH levels were significantly lower in the EB and HIGH DPN groups compared to the vehicle treated control group (*P *≤ 0.05). A trend for lower LH levels in the MID DPN group was also observed (*P *≤ 0.06). The overall pattern of serum LH levels was consistent with the pattern of GnRH and Fos co-labeling (Figure 2).

#### Density of Kiss-ir in the AVPV

**Figure 3:**
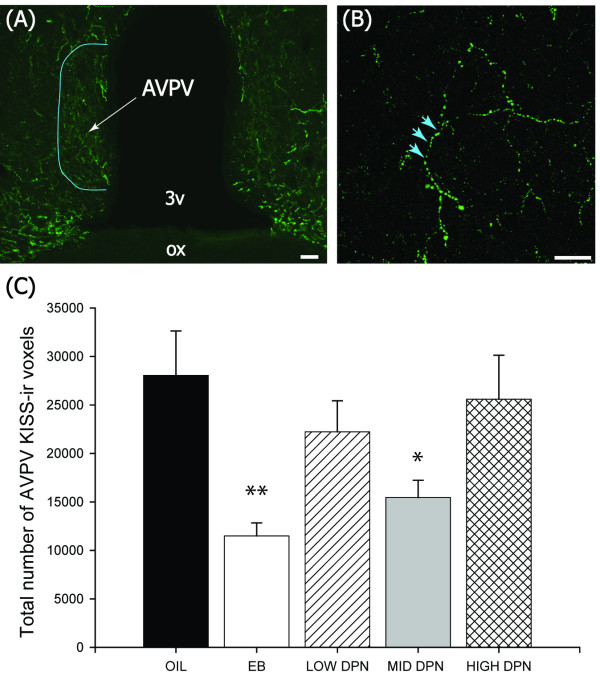
**Kisspeptin immunolabeling in the anterior hypothalamus**. Fluorescence photomicrographs showing Kiss immunoreactive (-ir) labeling in the AVPV of the hormone primed females (Experiment 1). **(A) **Kiss-ir was localized to extended lengths of fibers within and surrounding the AVPV. **(B) **Kiss-ir fibers (blue arrows) were readily visible and quantifiable at high magnification with the confocal microscope. The density of Kiss immunostaining was significantly lower in the EB and MID DPN (1 mg/kg) treated groups compared to the control animals. (3v = 3^rd ^ventricle, ox = optic chiasm, (A) Scale bar = 50 μm, (B) Scale bar = 25 μm, Means ± S.E.M., *P ≤ 0.05, **P ≤ 0.005).

Kiss-ir in the AVPV was primarily visualized as extended lengths of fibers and significantly affected by group (F(4,16 = 3.762, *P *≤ 0.02). As expected [4,20] the density of Kiss-ir fibers was significantly lower in the females neonatally exposed to EB (*P *≤ 0.005, n = 4) compared to the vehicle treated controls (Figure 3, n = 4). The effect of neonatal DPN exposure was dose dependent but the curve was u-shaped rather than linear with the most significant reduction in AVPV Kiss-ir occurring in the MID DPN (1 mg/kg) group (Figure 3; *P *≤ 0.03, n = 4). Kiss-ir fiber density was slightly lower than vehicle controls in the LOW DPN (*P *= 0.27, n = 4) and the HIGH DPN (*P *= 0.62) groups but the effect was not significant in either case.

#### Kiss-ir fibers and cells in the ARC

**Figure 4:**
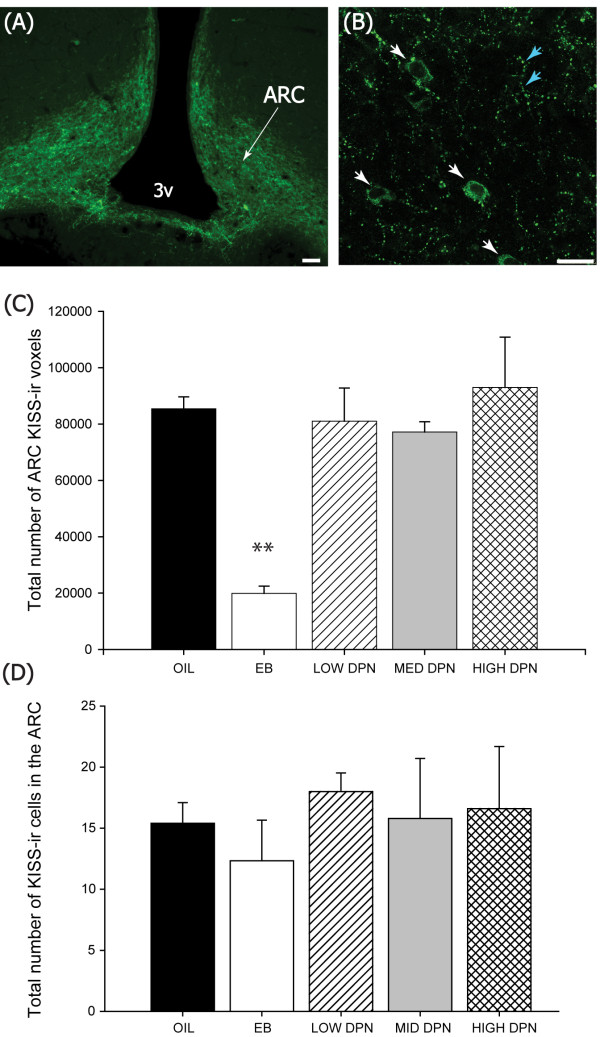
**Kisspeptin immunolabeling in the mediobasal hypothalamus**. Fluorescence photomicrographs showing Kiss immunoreactive (-ir) labeling in the ARC of the hormone primed females (Experiment 1). Kiss immunolabeling was considerably denser **(A) **than in the AVPV (see Figure 3) and localized to both fibers, **(B **blue arrows**) **as well as a small number of cell bodies that were often difficult to discern from the heavy fiber labeling (B, white arrows). **(C) **The density of Kiss-ir labeling was significantly lower in the females neonatally treated with EB but not DPN at any dose examined. **(D) **The number of Kiss-ir cell bodies did not statistically differ between groups. (3v = 3^rd ^ventricle, (A) Scale bar = 50 μm, (B) Scale bar = 25 μm, Means ± S.E.M., **P ≤ 0.001).

There was an overall effect of group (F(4,17 = 7.086; *P *≤ 0.002) on the density of Kiss-ir fibers in the ARC but this was entirely driven by the significantly lower levels in the EB treated females (Figure 4, n = 4) compared to the vehicle treated control females (n = 5, *P *≤ 0.001). DPN did not impact the density of Kiss-ir fibers in the ARC regardless of dose. Immunolabeled perikarya did not significantly differ between groups (F(4,17 = 0.130, *P *= 0.969).

### Experiment 2

#### Kiss1 mRNA expression in the AVPV

**Figure 5:**
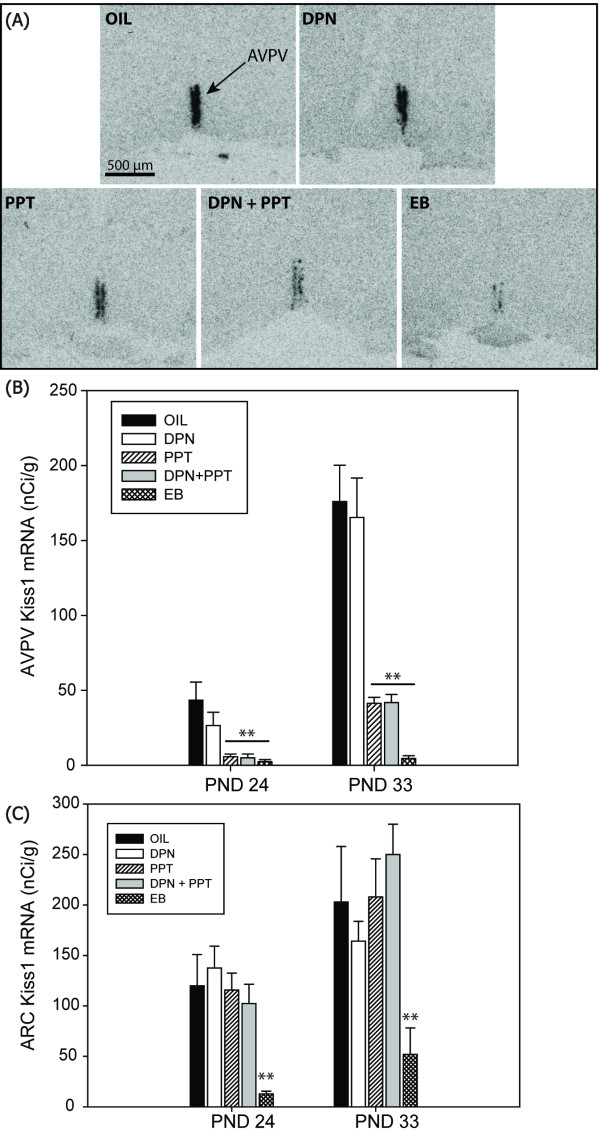
**Hypothalamic kisspeptin mRNA expression in peripubertal females**. **(A) **Representative autoradiographs depicting Kiss1 mRNA signal in the AVPV of periputertal females (Experiment 2). **(B) **Expression was significantly higher in the control (OIL) group compared to the PPT, DPN+PPT and EB groups at both PND24 and 33. **(C) **Within the ARC, expression was only significantly lower in the EB group compared to the OIL controls at both ages examined. Expression was not significantly altered in any of the other groups. (Scale bar = 500 μm, **P ≤ 0.005).

Labeling was readily discernable and evenly distributed throughout the entire AVPV of the PND 24 and 33 (Figure 5A) animals. Two-way ANOVA revealed a significant effect of age (F(1,49) = 64.009; *P *≤ 0.001) and exposure group (F(4,49) = 24.284; *P *≤ 0.001) as well as a significant interaction (F(4,49) = 10.074; *P *≤ 0.001) on Kiss1 mRNA levels in the AVPV (Figure 5B). As anticipated, levels increased with age in all groups except the EB group. Within the PND 24 animals, there was a significant effect of exposure (F(4,24) = 7.161; *P *≤ 0.001). Compared to vehicle controls, females exposed to EB, PPT or DPN+PPT had significantly lower Kiss1 mRNA levels (*P *≤ 0.001 in all cases). Levels in the DPN animals were also lower, but not significantly so (*P *= 0.08). These differences were even more pronounced on PND 33 (post-pubertal onset). Again there was a main effect of exposure group (F(4,25) = 20.083; *P *≤ 0.001) and the EB, PPT and DPN+PPT females had significantly lower Kiss1 mRNA levels compared to the control females (*P *≤ 0.001 in all cases). DPN exposed female Kiss1 mRNA levels were not significantly different from controls.

#### Kiss1 mRNA expression in the ARC

Labeling in the ARC was even more robust than in the AVPV and evenly distributed between the rostral and caudal borders. Two-way ANOVA revealed a significant effect of age (F(1,48) = 12.932; *P *≤ 0.001) and exposure group (F(4,48) = 5.516; *P *≤ 0.001) but no significant interaction on Kiss1 mRNA levels in the ARC (Figure 5C). On PND 24 there was a significant effect of exposure (F(4,24) = 3.731; *P *≤ 0.02) with only the EB group having significantly lower expression levels than the controls (*P *≤ 0.005). Similarly, on PND 33 there was also a significant effect of group (F(4,24) = 3.463; *P *≤ 0.023) and again only the EB exposed animals had lower expression compared to controls (*P *≤ 0.005).

## Discussion

Neonatal agonism of ERβ by DPN, at the three doses examined, produced only moderate disruption of the HPG axis. Physiological impacts included advanced vaginal opening and premature anestrus. A diminished capacity to elicit Fos labeling in GnRH neurons in response to hormone priming was observed, and although it corresponded with lower LH levels, this effect only reached statistical significance at the highest dose. Impacts on the kisspeptin system were minimal. Reduced Kiss-ir fiber density within the AVPV was only observed at the 1 mg/kg dose and no effects on ARC Kiss-ir were observed. Moreover, peripubertal Kiss1 mRNA levels were not significantly affected by DPN in the AVPV or ARC. In contrast, AVPV expression was significantly abrogated by EB or the ERα agonist PPT. These observations are consistent with what we have reported previously using the 1 mg/kg dose of DPN [4] and support the hypothesis that ERα plays a more substantial role than ERβ in the estrogen-dependent defeminization of hypothalamic steroid positive feedback signaling pathways during the neonatal critical period.

There was a dose dependent effect of neonatal DPN administration on DOV, a hallmark of puberty in the rat. Although all three doses significantly advanced pubertal onset, none resulted in a DOV as early as the animals exposed to EB. Body weight can be a confounding factor in the timing of pubertal onset, with heavier animals progressing through puberty earlier than lighter animals. This does not appear to have been the case here because there was no significant effect of exposure on body weight. Body weights were equivalent across the groups, and the heaviest animals were in the vehicle treated control group. The 1 mg/kg dose of DPN (MID DPN) advanced puberty by about four days, a shift that is considerably larger than what we have observed previously [4]. For the present study we used nearly twice as many animals so this difference may at least be partially attributable to the resulting increased statistical power or to other factors we cannot readily account for, such as time of year or quality of maternal care. The specific mechanisms by which estrogen exposure advances DOV remain unclear. Because exogenous administration of kisspeptin has been shown to advance puberty [63,64], one possibility is that early DOV results from the premature stimulation of GnRH neurons by kisspeptin. The present results are not consistent with this idea, however, because Kiss1 expression was not elevated in the PND 24 or PND 33 females that ultimately displayed early DOV. In contrast, AVPV levels were significantly decreased in EB exposed females, an effect more indicative of masculinization.

Neonatal agonism of ERβ also significantly curtailed the capacity to maintain a regular estrous cycle. The effect was dose dependent with animals in the group administered the highest dose generally entering a state of anestrus earlier than animals given the lower two doses. By PND 70, more than half of the females in the MID and LOW DPN groups were still cycling, at a point long after nearly all females neonatally exposed to EB or PPT become anestrus [4]. The observation that the administration of either PPT or DPN can limit the duration over which the estrous cycle progresses normally is consistent with results obtained in a prior study, using different ER selective agonists [65]. In that study, agonism of ERβ was found to have a more potent effect on estrous cyclicity than agonism of ERα, a result opposite to what we have observed. This could result from the binding and transcriptional properties of the selective agonists, or the timing of exposure. Collectively, however, it is evident from these findings that premature anestrus can result from neonatal exposure to estrogen agonists.

Even though selective agonism of ERβ was not as effective as EB (or PPT) in defeminizing steroid positive feedback on GnRH, it was not completely inconsequential, as the capacity to induce GnRH and Fos co-labeling was reduced by nearly half at the LOW and MID doses and by nearly two-thirds at the HIGH dose. Correspondingly lower LH secretion was also observed, but only at the highest dose. This dose dependent effect of DPN reveals that the capacity to generate GnRH and Fos co-labeling must be abrogated by at least half before appreciable disruption of gonadotropin secretion occurs. This is not entirely remarkable because there are numerous examples where neural cell loss or dysfunction must be substantial before physiological effects are observed. A notable example is Parkinson's disease, in which upwards of 60 to 70% of dopaminergic nigrostriatal neurons must be lost before motor symptoms manifest [66].

Although it is generally accepted that hormonal signals are largely conveyed to GnRH neurons by other hormone sensitive neurons within the hypothalamus, GnRH neurons constitutively express ERβ throughout development [67-69]. Therefore, it is at least theoretically possible that the partial impairment of steroid positive feedback on GnRH neurons resulted from the direct activation of ERβ on GnRH neurons themselves. Neonatal DPN exposure did not affect the number of GnRH neurons in the anterior hypothalamus, a finding which is consistent with other studies examining the impact of developmental exposure to estrogens or estrogen-like compounds on GnRH neurons [51,53,70], so this cannot be the mechanism by which GnRH neuronal activation was curtailed. An alternative possibility is that DPN acted on other ERβ expressing neurons within the hypothalamus. It is appealing to predict that DPN acted on AVPV Kiss-ir neurons directly, but this hypothesis may prove difficult to test. In the adult female rat, nearly all Kiss-ir neurons are found to be co-localized with either ERα or (to a lesser degree) ERβ [71] but it appears that neither Kiss1 mRNA nor Kiss-ir are detectable until approximately two weeks after birth [21,22,57]. It is likely that this neuronal population is present but quiescent during this time, but this remains to be clearly established. Thus, it is plausible that early life exposure alters the sex specific organization and function of this neuronal population. A biomarker for these neurons in early neonatal life would be needed to test this hypothesis. Consistent with this idea, however, we have recently shown that there are marked sex differences in ERα and ERβ mRNA expression in the neonatal rat AVPV [22] with females having higher levels of ERα, but males having higher ERβ levels than females, on the day of birth. This sex difference could at least partially account for why females appear to be more sensitive to the masculinizing influence of ERα agonists at this age. It is also possible that ERβ-expressing neurons with afferents to AVPV Kiss neurons, rather than Kiss neurons themselves, are mediating the decrease in Kiss-ir and GnRH activity. If this is the case, it remains to be determined what those neurons might be. In addition, selective activation of ERβ might have impacted the expression of ERα in the AVPV, and our subsequent observations, therefore, reflect changes in ERα activity [72]. It has long been hypothesized that each ER subtype modulates the expression and activity of the other in a region specific manner [73-75] and this balance may have been disrupted by DPN administration. Regardless of how or where DPN is stimulating ERβ activity, our data show that the net result is only a marginal decrease in AVPV Kiss-ir and GnRH neuronal activation in the adult female.

The impact of DPN on the organization of Kiss-ir in the female AVPV was dose dependent but, unlike the other endpoints we examined, the dose response curve was u-shaped rather than linear. Maximal reduction was observed at the middle (1 mg/kg) dose. In contrast, the suppression of GnRH activation by DPN was approximately equivalent across all three DPN doses and thus consistent with Kiss1 mRNA levels (Figure 5), but not concomitant with the u-shaped dose effect on AVPV Kiss-ir levels. It is not readily clear why this one endpoint, and not the others, produced a u-shaped response curve or what the functional significance of it might be. It may simply be a spurious observation, a possibility consistent with the failure to find an appreciable effect of DPN on AVPV Kiss1 expression in peripubertal females. Notably this nonmonotonic type of dose response curve is not atypical for estrogen or estrogen agonists. For example, a biphasic effect of estradiol on terminal end bud formation in the mammary gland and prostate hyperplasia have been described previously [76,77]. A definitive mechanism to explain this phenomenon is lacking, but several hypotheses have been put forth, including the differential regulation of hormone receptors with dose, and the overlap of two distinct mechanisms of action [78]. For the present study, both hypotheses are plausible. In the MID DPN females, LH levels are lower but not significantly so, indicating that the lower numbers of Kiss-ir fibers may have functional significance. Normal levels in the HIGH DPN females may reflect a method by which the system is attempting to compensate for decreased GnRH sensitivity to steroid hormone stimulation. Further studies incorporating serial sampling of LH are needed to explore these possibilities.

Kiss fiber density and mRNA expression was reduced in the ARC of the EB but not the DPN exposed animals, an effect which was not completely unexpected given that ERβ does not appear to be present in the neonate [22] or adult female ARC [79,80]. Many of these fibers likely originate from the AVPV population [81]. Collectively, the data presented here suggest that ERβ agonism does not alter the density of this plexus. More perplexing, however, Kiss1 mRNA expression was not affected by neonatal PPT exposure nor the combination of DPN and PPT. The significant effect of EB is consistent with what we have seen previously, but remains somewhat surprising given that neither the number of Kiss neurons nor the quantity of Kiss1 expression in the ARC have been found to be sexually dimorphic in rats [26]. It remains to be determined why neonatal EB administration has such a profound effect on a population of neurons that do not appear to be sexually dimorphic or what the functional significance of this might be. It also remains to be established why the co-administration of DPN and PPT did not recapitulate the effect of EB. It is possible that EB is acting at the level of the membrane or via a "non-classical" pathway to induce the effect [82,83].

As discussed previously, the doses of DPN chosen for this study were selected to bracket the dose of 1 mg/kg that we and others have used before [4,33]. Because DPN has only a 70-fold greater relative binding affinity for ERβ than ERα [32], at high doses it may also begin to bind and activate ERα. Although the highest dose used for the present studies (2 mg/kg) has been successfully employed by others with no evidence of ERα agonsim [36], it is possible that agonism of ERα occurred to some degree. Partial agonism of ERα could explain why the HIGH DPN females had earlier puberty and lost their estrous cycle more quickly than those in the other two DPN groups, and why GnRH activation was somewhat lower at this dose compared to the other two. Even if agonism of ERα occurs at this dose, from the totality of the data it is evident that agonism of ERβ alone was insufficient to affect any of the endpoints observed to the same degree as EB.

## Conclusion

In summary, neonatal estrogen defeminizes the hypothalamus such that the coordination of steroid positive feedback on GnRH neurons and, consequently, the maintenance of a regular ovulatory cycle are curtailed. The AVPV population of Kiss neurons is also sexually differentiated by neonatal estrogen and believed to be pivotal for initiating the pre-ovulatory surge of GnRH [12,25]. Here we have shown that neonatal administration of the ERβ agonist DPN does not affect the ontogeny of Kiss1 expression across peripuberty or induce functional Kiss/GnRH signaling disruptions, resulting in abrogated LH release, at a dose below 2 mg/kg. These results indicate that selective agonism of ERβ is not sufficient to completely achieve the male-typical HPG phenotype seen with either EB or PPT.

## Abbreviations

ARC: arcuate nucleus; AVPV: anteroventral periventricular nucleus; DPN: diarylpropionitrile; DOV: day of vaginal opening; ERα: estrogen receptor alpha; ERβ: estrogen receptor beta; EB: estradiol benzoate; GnRH: gonadotropin releasing hormone; HPG: hypothalamic pituitary gonadal axis; IHC: immunohistochemistry; -ir: immunoreactive; ISH: *in situ *hybridization; Kiss: kisspeptin; LH: luteinizing hormone; LSD: Fisher's Least Significant Difference; MPOA: medial preoptic area; NCSU: North Carolina State University; OVLT: organum vasculosum of the lamina terminalis; OVX: ovariectomize; PND: postnatal day; PPT: propyl-pyrazole-triol; RIA: radioimmunoassay; RT-PCR: reverse transcription-polymerase chain reaction.

## Competing interests

The authors declare that they have no competing interests.

## Authors' contributions

SML was responsible for all dosing and animal handling for both experiments. KLT and JAM performed all of the ovariectomies, completed the perfusions, prepared the resulting brain tissue and performed the immunolabeling and quantification with the assistance of SML. KAM and SML prepared the tissue for Experiment 2 and performed the *in situ *hybridization. KLT quantified optical density. HBP and SML designed the experiments and prepared the figures. HBP prepared the manuscript and the remaining authors edited and proofed it. All of the authors have read and approved the final manuscript.
